# The Human Melanoma Side Population Displays Molecular and Functional Characteristics of Enriched Chemoresistance and Tumorigenesis

**DOI:** 10.1371/journal.pone.0076550

**Published:** 2013-10-03

**Authors:** Jasper Wouters, Marguerite Stas, Lies Gremeaux, Olivier Govaere, Anke Van den broeck, Hannelore Maes, Patrizia Agostinis, Tania Roskams, Joost J. van den Oord, Hugo Vankelecom

**Affiliations:** 1 Translational Cell & Tissue Research, Dept. of Imaging and Pathology, University of Leuven (KU Leuven), Leuven, Belgium; 2 Research Unit of Stem Cell Research (Lab. of Tissue Plasticity), Cluster Stem Cell Biology and Embryology, Dept. of Development and Regeneration, University of Leuven (KU Leuven), Leuven, Belgium; 3 Surgical Oncology, Dept. of Oncology, University of Leuven (KU Leuven), Leuven, Belgium; 4 Abdominal Surgical Oncology, Dept. of Oncology, University of Leuven (KU Leuven), Leuven, Belgium; 5 Lab. of Cell Death Research & Therapy, Dept. of Cellular and Molecular Medicine, University of Leuven (KU Leuven), Leuven, Belgium; University of Frankfurt - University Hospital Frankfurt, Germany

## Abstract

Melanoma remains the most lethal skin cancer, mainly because of high resistance to therapy. Side population (SP) cells are found in many types of cancer and are usually enriched in therapy-resistant as well as tumorigenic cells. Here, we identified a Hoechst dye-effluxing SP in a large series of human melanoma samples representing different progression phases. The SP size did not change with disease stage but was correlated with the prognostic “Breslow’s depth” in the primary (cutaneous) tumors. When injected into immunodeficient mice, the SP generated larger tumors than the bulk “main population” (MP) melanoma cells in two consecutive generations, and showed tumorigenic capacity at lower cell numbers than the MP. In addition, the SP reconstituted the heterogeneous composition of the human A375 melanoma cell line, and its clonogenic activity was 2.5-fold higher than that of the MP. Gene-expression analysis revealed upregulated expression in the melanoma SP (*versus* the MP) of genes associated with chemoresistance and anti-apoptosis. Consistent with these molecular characteristics, the SP increased in proportion when A375 cells were exposed to the melanoma standard chemotherapeutic agent dacarbazine, and to the aggravating condition of hypoxia. In addition, the SP showed enhanced expression of genes related to cell invasion and migration, as well as to putative (melanoma) cancer stem cells (CSC) including *ABCB1* and *JARID1B*. ABCB1 immunoreactivity was detected in a number of tumor cells in human melanomas, and in particular in clusters at the invasive front of the primary tumors. Together, our findings support that the human melanoma SP is enriched in tumorigenic and chemoresistant capacity, considered key characteristics of CSC. The melanoma SP may therefore represent an interesting therapeutic target.

## Introduction

Cutaneous malignant melanoma is the most lethal form of skin cancer representing more than 75% of skin cancer-related deaths [1]. In advanced (metastasized) stages, melanoma is dismal and almost impossible to treat resulting in a 5-year survival rate of only 15% [1]. Resistance to chemotherapy is a major cause of treatment failure, and better knowledge of the melanoma chemoresistant cells can open the way to more efficient therapies.

In several types of cancer, the so-called side population (SP) enriches for chemoresistant cells [2–4]. SP cells are identified by their ability to efficiently efflux Hoechst dye through the activity of multidrug transporters like ABCB1 (also known as P-glycoprotein, P-GP or Multidrug Resistance Protein 1, MDR1), ABCG2 (or Breast Cancer Resistance Protein 1, BCRP1), ABCB5 and/or ABCC1 (or Multidrug Resistance-associated Protein 1, MRP1) [2–4]. Because of this dye expulsion, the SP is portrayed as a side-branch of Hoechst^low^ cells in dual-wavelength flow cytometry (FACS) [2–6]. Interestingly, ABCB1, ABCG2, ABCB5 and ABCC1 have been found important in melanoma chemoresistance [7–10]. Moreover, SP phenotype and chemoresistance are proposed characteristics of cancer stem cells (CSC), a subpopulation within the tumor that holds the highest capacity to drive growth and progression (with invasion and metastasis) of the tumor ( [2–4] and reviewed in 11–13). Recent studies clearly support the existence of CSC in cancers, their chemoresistant nature and functional relevance [14–17]. Interestingly, one of the multidrug transporters - ABCB5 -, has been put forward as a marker of melanoma CSC [18]. Although this and additional factors (like JARID1B, NES, NGFR/CD271, SOX10, TDGF1/CRIPTO) have been proposed to mark melanoma CSC (reviewed in 13), the existence of CSC in melanoma tumors remains contentious.

Previous studies have identified a SP in melanoma cell lines propagated *in vitro* and have provided arguments supporting a chemoresistant and CSC-like phenotype including tumorigenic potential and expression of NES or *JARID1B* [19–22]. Very recently, Luo et al. reported the presence of a SP in a small number of clinical human melanoma tumors (n=8), analyzed whole-genome expression of metastasized samples (lymph node) after expansion in immunodeficient mice (n=2), and found the SP to be more resistant to paclitaxel and temozolomide than the non-SP cells [10]. In the present study, we analyzed a larger series of human melanoma specimens covering a wide range of progression phases, explored its prognostic potential, determined genome-wide expression in primary melanomas directly from the patient, and tested resistance to dacarbazine, still the most commonly used single-agent chemotherapeutic in advanced-melanoma therapy [23]. In addition, resistance to hypoxia, and tumorigenic and clonogenic potential were investigated. Together, our analyses point toward enrichment of the human melanoma SP in chemoresistant and tumorigenic activity. 

## Results

### Human melanoma contains a side population

In a recent study by Luo et al., a Hoechst dye-excluding side population (SP) was identified in 8 clinical melanoma samples [10]. Meanwhile, we analyzed the SP in a larger series of patient melanoma tumors covering different progression phases (n=38; Table S1 in File S1), and examined the correlation with disease stage and tumor thickness (Breslow’s depth), a strong prognostic factor and key parameter in melanoma staging [1,24].

**Figure 1:**
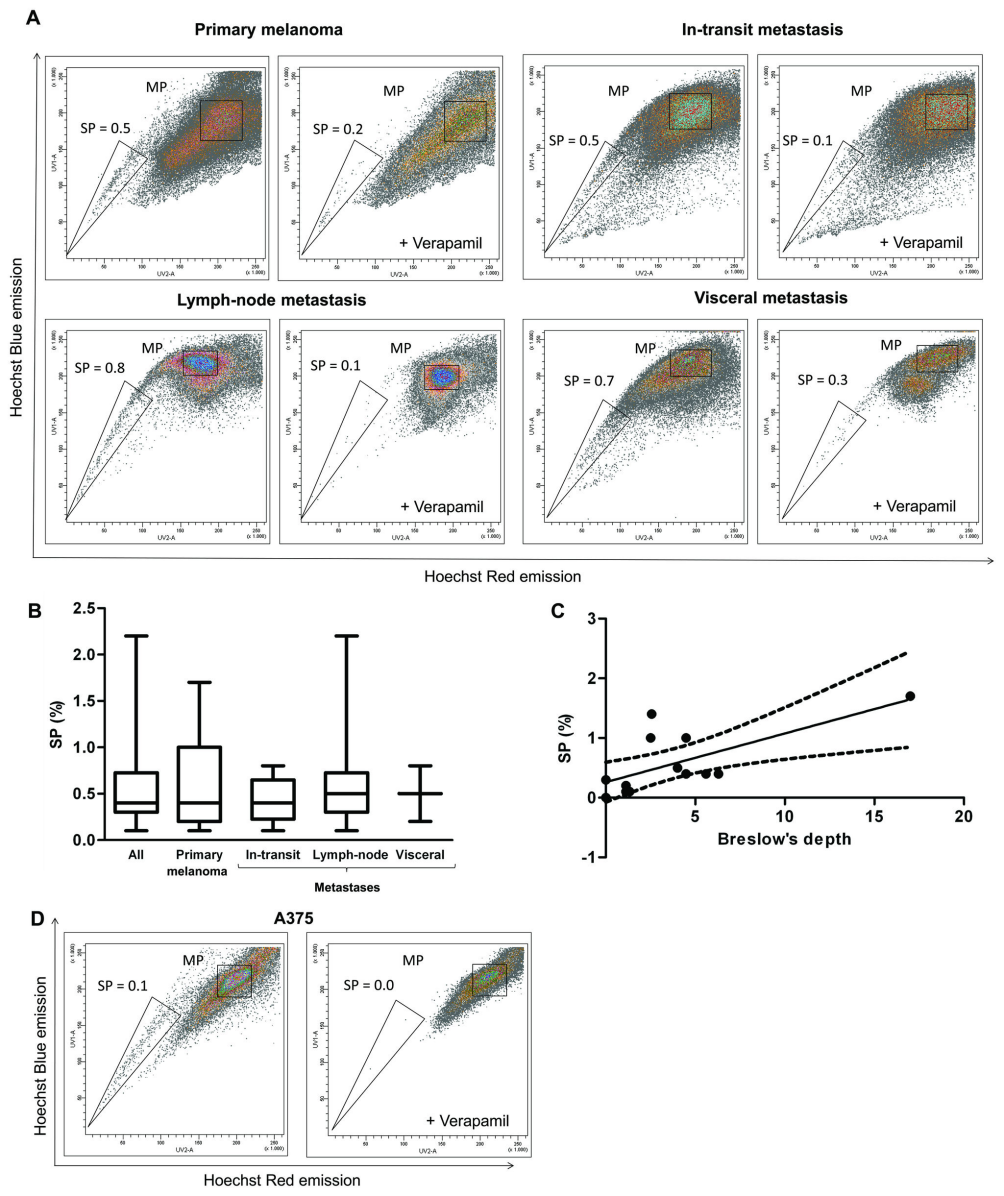
The side population (SP) in human melanoma of various progression phases. A) Representative examples of FACS density plots of Hoechst-incubated cells from primary melanoma (cutaneous malignant form; top left), in-transit metastasis (top right), metastasis to a regional lymph node (bottom left) and visceral metastasis (bottom right) with indication of SP percentage, and the corresponding controls with verapamil. B) Boxplot of the SP proportions from all human melanoma samples taken together (All, n=38), and from the different progression phases as indicated (primary melanomas, n=13; in-transit metastases, n=8; regional lymph-node metastases, n=14; visceral metastases, n=3). C) SP proportion in primary (cutaneous malignant) melanomas as outlined against tumor’s thickness (Breslow’s depth). A significant correlation is observed (Spearman r=0.68; p=0.01; dashed line, 95% confidence interval of the best-fit line). D) Representative example of FACS density plots of Hoechst-incubated cells from the human malignant melanoma cell line A375 with indication of SP percentage, and the corresponding verapamil control (n=18).

A SP was detected in all melanoma samples analyzed, representing 0.1-2.2% of the viable tumor cells (median SP: 0.4%; Figure 1A-B). Verapamil, an inhibitor of efflux pumps, strongly reduced the SP percentage, thereby confirming the SP phenotype (Figure 1A). The SP proportion did not significantly change between the various melanoma progression stages (Figure 1A-B); primary melanomas (cutaneous malignant form), in-transit metastases, lymph-node metastases and visceral metastases harbor a median SP of 0.4% (n=13), 0.4% (n=8), 0.5% (n=14) and 0.5% (n=3), respectively. Of note, a SP was not detected in pre-malignant nevus (data not shown). Interestingly, the SP proportion in the primary melanomas was found to correlate with Breslow’s depth (Figure 1C; p=0.01).

Because of the restricted availability of patient-derived melanoma tissue, we also used the human malignant melanoma A375 cell line [25] for further extended SP characterization. In the cell line we identified a small but clear SP (Figure 1D; range SP: 0.1-0.3%; median: 0.1%; n=18).

### The melanoma SP is enriched in tumorigenic activity

In several types of cancer, the SP is enriched in cells that are more tumorigenic than the other cancer cells and that can regrow the tumor [3,26–28]. These cells are generically designated as CSC.

**Figure 2:**
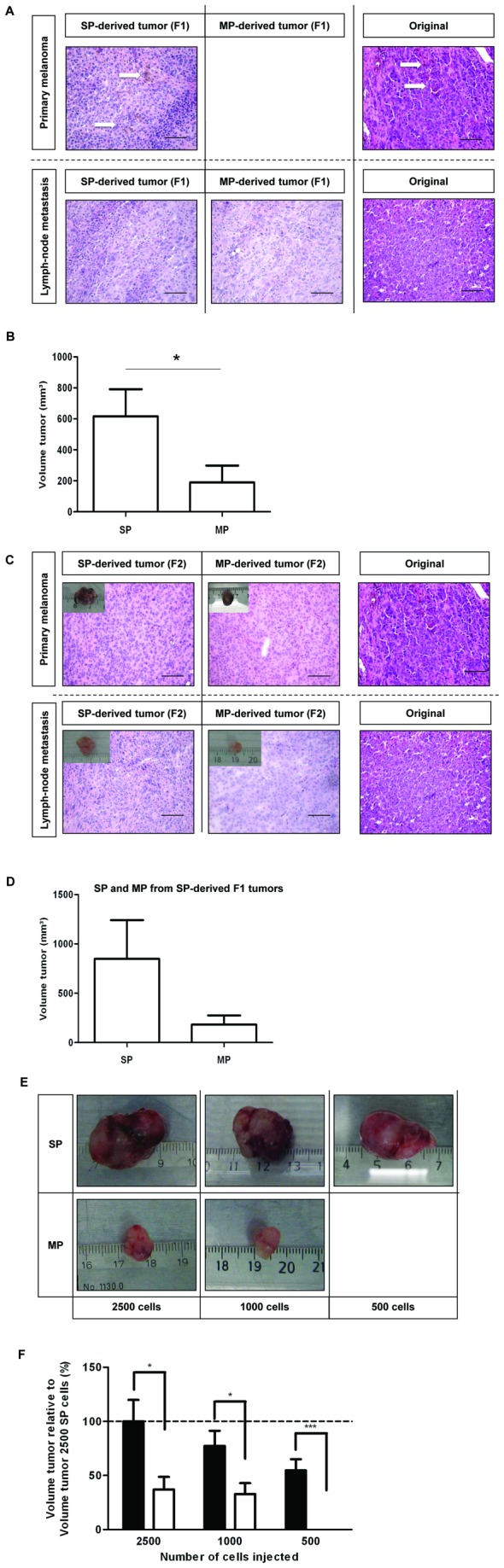
*In vivo* tumorigenic activity of the melanoma SP. A) Sections (H&E staining) of the xenograft tumors developed in SCID mice 18 weeks after sc injection of SP and MP cells from a primary melanoma (pigmented, see arrows) (upper row) and from a lymph-node metastasis (lower row), together with sections of the original tumors (scale bar, 150µm). B) Volume of the xenograft tumors grown in SCID mice 18 weeks after sc injection of the melanoma SP and MP cells (10,000-50,000 cells). Bars represent mean ± SEM (n=6). *, p<0.05. C) Photographs and sections (H&E staining) of the xenograft tumors developed in SCID mice 18-32 weeks after sc injection of SP and MP cells from the SP-derived first-generation xenograft tumors (F1, see A), together with sections of the original tumors (scale bar, 150µm). Again note the pigmentation (upper row) as clear from the picture of the dissected tumor. D) Volume of the F2 xenograft tumors grown in SCID mice 32 weeks after sc injection of the SP and MP cells from the SP-derived F1 tumors (10,000 SP cells, 50,000 MP cells). Bars represent mean ± SEM (n=2). E) Tumors grown in SCID mice 5-7 weeks after sc injection of 2500, 1000 and 500 SP cells (upper row) or MP cells (lower row) from the A375 melanoma cell line (n=3). Representative examples are shown. F) Summary of tumor volume (relative to volume of the tumors grown from 2500 SP cells) after sc injection of the indicated number of A375 SP cells (black bar) and MP cells (white bar). Bars represent mean ± SEM (n=3). *, p<0.05; ***, p<0.001.

We selected two melanoma progression phases (i.e. primary and lymph-node metastasis) from which to assess the *in vivo* tumorigenic activity of SP cells. Because primary melanomas were typically too small to sort a sufficient number of cells, they were first expanded in immunodeficient (SCID) mice. SP and bulk tumor “main population” (MP) cells were then sorted and 10,000 cells subcutaneously (sc) injected into SCID mice. The SP generated a new tumor in every experiment (n=3) whereas the MP produced a tumor at lower frequency (1 from n=3). To explore tumorigenic activity of a further melanoma progression stage and at the same time enabling the assessment of clinical melanoma without intervening expansion in a mouse, SP and MP cells were sorted from a larger lymph-node metastasis and sc transplanted into SCID mice. High cell numbers of SP and MP (50,000) both generated tumors at a similar pace, with visible appearance after 6-7 weeks. The re-formed tumors from both primary melanomas and lymph-node metastasis were histologically comparable to the original human lesions (Figure 2A). In particular, the SP from a pigmented melanoma generated a pigmented tumor (*arrows* in Figure 2A; no tumor from the MP). Of note, the tumors grown from the SP expanded more than from the MP, i.e. they were larger at the time point of analysis (18 weeks after injection; Figure 2B).

To investigate whether tumorigenic capacity is sustained, we again sorted the SP and MP cells from the SP-derived first-generation xenograft tumors (F1, see Figure 2A). Injection of 10,000 SP cells resulted in a visible tumor (F2) as early as 10 weeks after injection (n=2). In contrast, MP cells – injected at even 5-fold higher numbers (50,000) -, induced palpable F2 tumors only after 18-22 weeks. The F2 xenograft tumors again were histologically comparable to the original tumors (Figure 2C). In addition, the SP-derived F2 tumors were larger than the MP-derived tumors as analyzed at a late time point (32 weeks after injection; Figure 2C-D).

Together, the data above provide support that the melanoma SP contains higher tumorigenic activity than the MP.

**Figure 3:**
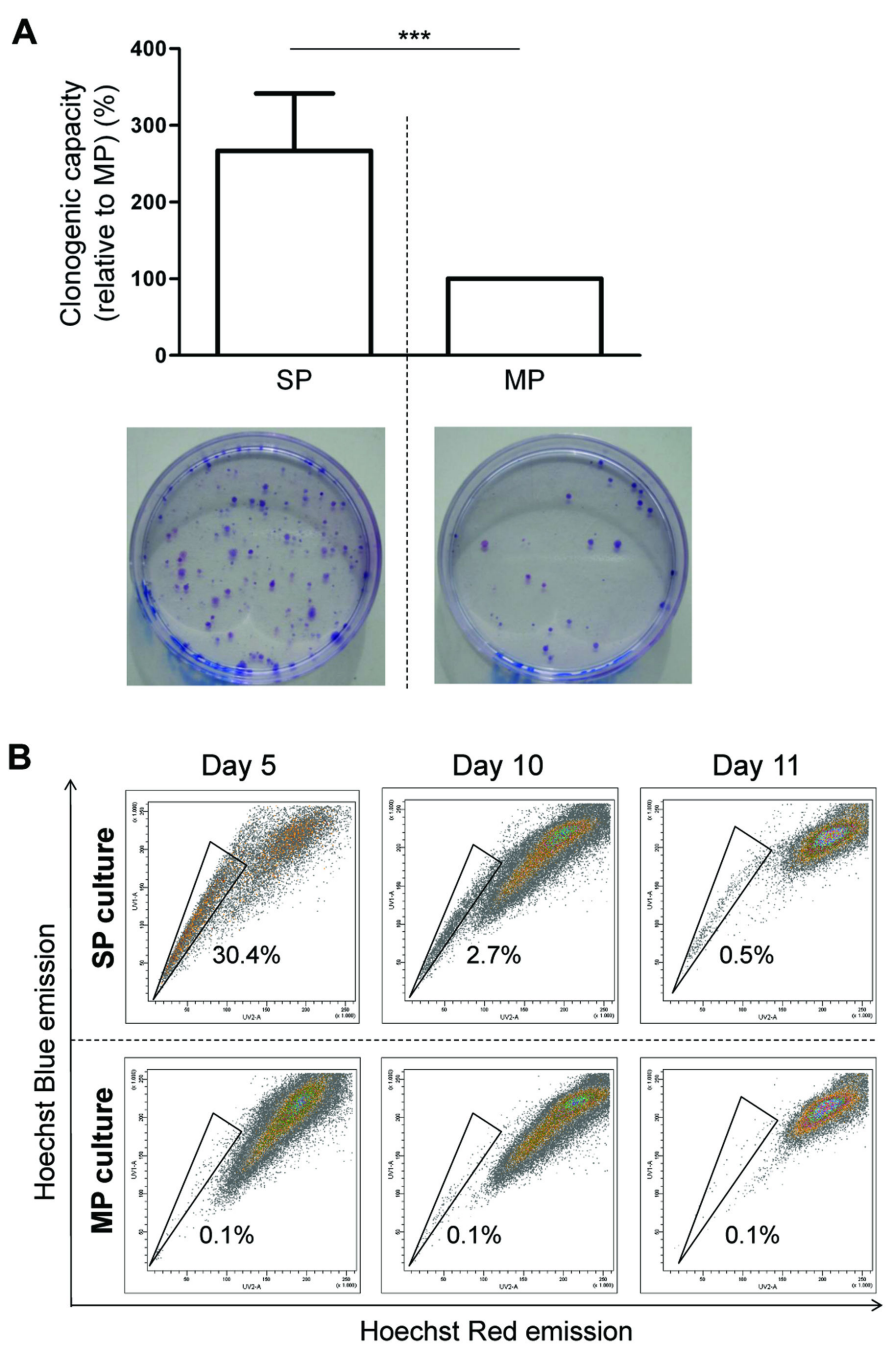
Clonogenic and reconstituting capacity of the melanoma SP. A) Clonogenic capacity of A375 SP cells (relative to MP cells) as assessed at day 9 after seeding at low density. Representative examples of colony-forming activity by SP (bottom left) and MP (bottom right) are shown. Bars represent mean ± SEM (n=3). ***, p<0.001 *versus* MP. B) SP analysis (representative FACS density plots) 5, 10 and 11 days after seeding A375 SP cells (upper row) and MP cells (lower row) in standard culture medium (n=4 except for day 11 where n=2; numbers indicate the mean SP proportion).

Finally, to examine tumorigenic activity in conditions that are more permissive to standardization, we analyzed tumorigenesis by SP and MP cells from the A375 cell line and at the same time tested smaller numbers of cells. After sorting by FACS, 2500, 1000 and 500 SP or MP cells were sc transplanted into SCID mice. Irrespective of cell numbers injected, SP-derived tumors were significantly larger than the tumors grown from the MP (Figure 2E-F; n=3). Moreover, at the lowest number of cells tested, the MP became incapable of generating tumors whereas the SP remained tumorigenic. In agreement with this higher *in vivo* tumorigenic activity, SP cells (from A375) generated more colonies (~2.5-fold) than MP cells when living (propidium iodide-negative) cells were seeded *in vitro* at equal low density (Figure 3A). Furthermore, the A375 SP was able to gradually reconstitute the cell line’s heterogeneity when seeded in culture, i.e. to re-generate the MP with concurrent decrease of the SP proportion (Figure 3B). Of note, the MP still contained some SP cells (~0.1%) after sorting which did not change during culture (Figure 3B).

Taken together, the melanoma SP is enriched in cells with higher clonogenic and tumorigenic activity than the MP, and seems able to reconstitute the tumor’s heterogeneity *in vivo* and *in vitro*. These findings support the idea that the SP encompasses melanoma CSC or CSC-like cells.

### Gene expression profiling of melanoma SP

In the recent study by Luo et al., SP gene expression was determined starting from xenograft tumors of lymph-node metastases (n=2) [10]. Here, we explored whole-genome expression in SP (*versus* MP) from primary melanomas obtained directly from the patient (n=4). Microarray analysis revealed 462 differentially expressed genes (≥1.5-fold up or down, p<0.05; see Table S2 in File S1). Of these genes, 264 were differentially expressed in at least 3 of the 4 individual samples (*bold* in Table S2 in File S1), corresponding to 251 human ENSEMBL IDs of which 142 and 109 genes were up- and downregulated, respectively, in the SP compared to the MP. The upregulated expression of a few genes (*DDX17*, *MARCH8*, *TNRC6A*) was confirmed by RT-qPCR (using the limited residual RNA/cDNA of 2 of the microarrayed melanoma samples; Figure S1A in File S2). The 251 differentially expressed genes were submitted to Gene Set Enrichment Analysis (GSEA) which revealed a significant overlap of the genes upregulated in the melanoma SP with gene sets upregulated in other cancers such as colon carcinoma (as compared to normal mucosa [29]) and bladder cancer with high recurrence rate (*versus* low recurrence rate [30]) (Table S3 in File S1). In addition, a significant overlap of genes downregulated in the melanoma SP was observed with gene sets downregulated in the breast cancer cell line MCF7 SP cells (*versus* the MP cells) [31], of quiescent cells of chronic myeloid leukemia (as compared to proliferating cells [32]) and of melanomas from patients that develop distant metastases within 4 years (as compared to non-metastasizing melanomas [33]) (Table S4 in File S1). Visualization of the interaction network of SP-upregulated genes by STRING analysis revealed that genes involved in regulation of apoptosis (e.g. *BAG4, CASP2, DDX17, IGF1* and *MDM2*), chemoresistance (e.g. *IGF1, IGF1R, MAPK14* and *MDM2*), and epithelial-mesenchymal transition (*MDM2*, *SETD8* and *TWIST1*) occupy a central position (Figure S1B in File S2). A further detailed and more focused analysis of a selection of genes of the microarray expression data revealed upregulated expression (≥1.5-fold in half or more of the 4 tumors, p<0.05) in the SP of genes implicated in “cancer stemness”, chemoresistance, anti-apoptosis, and cell invasion and migration; and downregulated expression of cell adherence and pro-apoptotic genes (Table 1 and Table S5 in File S1).

**Table 1:** Selection of functionally interesting genes differentially expressed between the SP and MP of primary melanomas.

**GeneName^1^**	**Fold^2^**	**p-value**	**Gene Function**
*DDX17*	4.85	0.004	anti-apoptosis; migration
*PDE4D*	2.90	0.030	invasion; metastasis
*HIPK1*	2.73	0.008	stemness
*PPID*	2.51	0.034	anti-apoptosis
*YWHAE*	2.27	0.003	tumorigenesis; metastasis
*ASB11*	2.18	0.019	stemness
*SLC18A1*	2.12	0.029	therapy resistance
*TRIM23*	2.12	0.019	metastasis; tumorigenesis
*SOCS3*	2.11	0.033	stemness; anti-apoptosis
*ZNF420*	2.05	0.049	anti-apoptosis
*MAPK14*	1.93	0.016	metastasis; stemness
*MDM2*	1.83	0.025	therapy resistance; anti-apoptosis
*HOXC10*	1.81	0.035	invasion
*IGF1R*	1.74	0.029	anti-apoptosis; migration
*BAG4*	1.71	0.040	anti-apoptosis
*SGK*	1.70	0.035	metastasis
*MFGE8*	1.70	0.042	tumorigenesis; metastasis
*PAK6*	1.67	0.020	migration; therapy resistance
*TP53RK*	1.67	0.047	anti-apoptosis
*NAV2*	1.61	0.014	migration
*IGF2BP1*	1.60	0.029	migration; therapy resistance
*MAPRE2*	1.59	0.016	invasion
*HSPA8*	1.59	0.005	metastasis
*IGF1*	1.59	0.023	anti-apoptosis; invasion; stemness
*TWIST1*	1.58	0.014	invasion; migration; metastasis; therapy resistance; stemness
*CRYAA*	1.58	0.046	anti-apoptosis
*ZFX*	1.57	0.015	anti-apoptosis
*SETD8*	1.55	0.008	metastasis
*ERAS*	1.55	0.018	stemness; therapy resistance; tumorigenesis
*BOK*	0.67	0.039	pro-apoptosis
*ERP29*	0.66	0.050	inhibition of migration; of invasion and of tumorigenesis
*PSMA7*	0.65	0.034	inhibition of migration and of tumorigenesis
*NRSN2*	0.65	0.010	tumor suppression
*APITD1*	0.64	0.026	tumor suppression
*LRRC4*	0.64	0.006	inhibition of invasion and of tumorigenesis
*SEC 14L2*	0.64	0.007	tumor suppression
*SCOTIN*	0.63	0.014	pro-apoptosis
*PRDM5*	0.57	0.048	tumor suppression
*MAGED1*	0.55	0.043	inhibition of invasion; of migration and of metastasis
*CLDN1*	0.55	0.027	inhibition of metastasis
*BMP7*	0.52	0.003	inhibition of metastasis and of stemness
*PTPRD*	0.31	0.019	tumor suppression

1 Genes related to “stemness”/CSC, therapy resistance, apoptosis, metastasis, and cell adherence, invasion and migration, differentially expressed between SP and MP in ≥half of the 4 primary melanomas as analyzed by microarray (≥1.5-fold, p<0.05).

2 Fold up- or downregulation in the SP *versus* MP.

**Figure 4:**
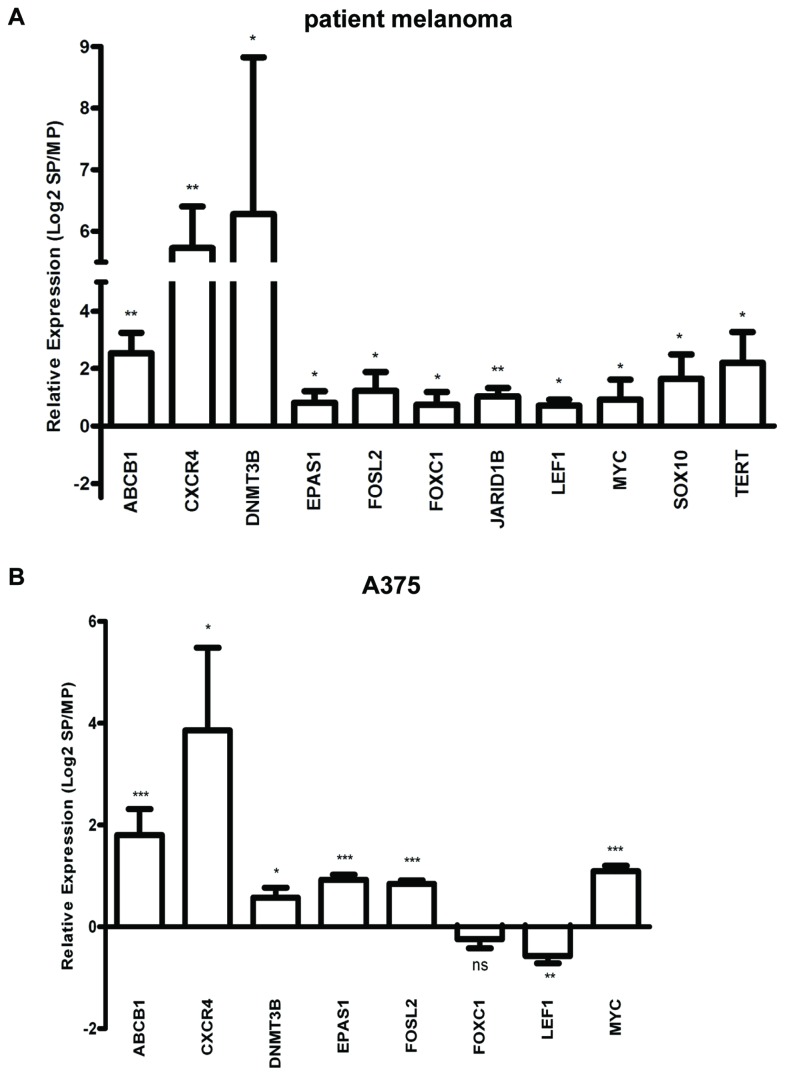
Gene expression in melanoma SP as analyzed by RT-qPCR. A) Expression ratios of the indicated genes in the SP *versus* the MP from patient melanomas. Bars represent mean ± SEM (n= 3 primary melanomas and 4 melanoma metastases). *, p<0.05; **, p<0.01. B) Expression ratios of the indicated genes in the SP *versus* the MP from the A375 cell line. Bars represent mean ± SEM (n=4). *, p<0.05; **, p<0.01; ***, p<0.001.

The SP is considered as a subpopulation that can efflux drugs, and that is enriched in candidate CSC in several cancers [3,4,26–28]. The higher tumorigenic activity as described above supports a CSC-like phenotype of the SP also in melanoma. To further investigate the drug-effluxing and CSC-like character, we analyzed an additional and larger set of melanoma samples (primary, n=3; metastases, n=4), obtained directly from the patients, by RT-qPCR for expression of ABC multidrug transporters and of CSC-associated markers (see Table S6 in File S1). Compared to the MP, SP cells showed a significantly higher expression of *ABCB1, CXCR4, DNMT3B, EPAS1* (*or hypoxia-inducible factor 2A, HIF2A*)*, FOSL2, FOXC1, JARID1B, LEF1, MYC, SOX10* and *TERT* (Figure 4A). Amongst these, *ABCB1, DNMT3B, EPAS1, JARID1B* and *TERT* have previously been designated as melanoma CSC(-associated) markers ( [8,22] and reviewed in 13). Of note, these transcripts were not revealed as significantly upregulated in the microarray analysis, which may be accounted for by the limited number of samples (n=4), causing low statistical power and differences in expression levels that may have been missed (i.e. not revealed as significant). Indeed, a number of the genes including *ABCB1* and *JARID1B* showed similar folds of upregulation in the microarray analysis, but the differences were not statistically significant (see Table S7 in File S1).

Furthermore, in the RT-qPCR analysis, *ABCG2, KLF4, POU5F1/OCT4, SNAI2* and *TDGF1* displayed upregulated expression, but also not statistically significant (Figure S2 in File S2). Other proposed CSC-associated (melanoma) markers like *ABCB5, CD44, MAGE-C2, NANOG, NES, NGFR* and *SOX2* did not show different expression levels in SP and MP (Figure S2 in File S2). Of note, expression of *ABCB5* was higher in the SP than the MP in 3 out of the 7 tumors (0.5- to 7.8-fold), and lower in the 4 other tumors (-1.7- to -9-fold), making the overall difference statistically non-significant. Finally, a selection of these factors was further validated using the A375 cell line (Figure 4B). The majority (except *FOXC1* and *LEF1*) was also found upregulated in the SP of the melanoma cell line.

### The melanoma SP shows resistance to dacarbazine chemotherapy and to hypoxia

**Figure 5:**
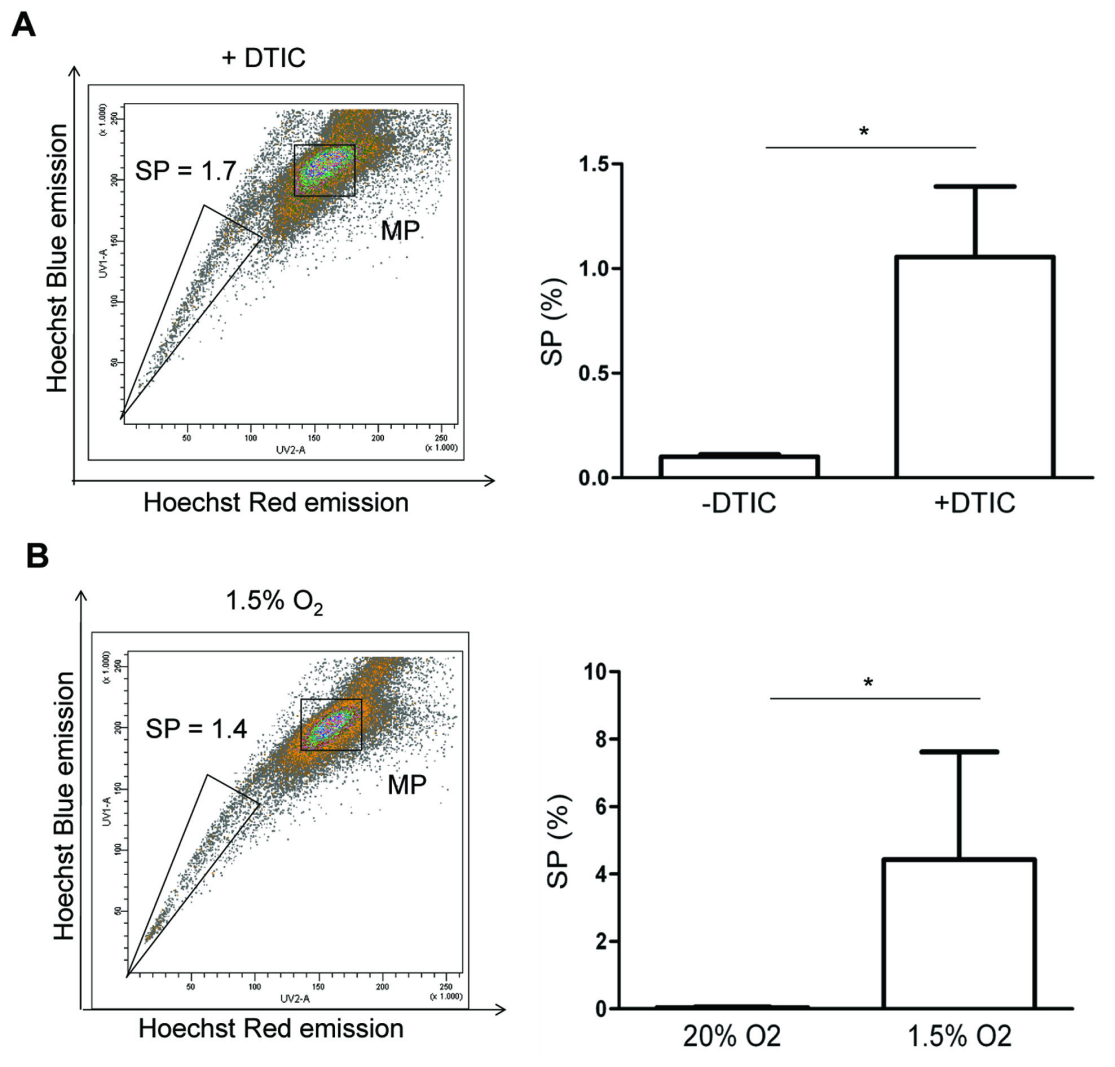
The melanoma SP after exposure to dacarbazine and hypoxia. A) Representative example of FACS density plots of Hoechst-incubated A375 cells treated with dacarbazine (+DTIC; 150µg/ml for 3 days) (left; verapamil control not shown) and summary of the SP proportions (right). Bars represent mean ± SEM (n=3). *, p<0.05 *versus* control (–DTIC). B) Representative example of FACS density plots of Hoechst-incubated A375 cells cultured in hypoxic conditions (1.5% O_2_ 3 days) (left; verapamil control not shown) and summary of the SP proportions (right). Bars represent mean ± SEM (n=3). *, p<0.05 *versus* standard cell-culture condition (20% O_2_).

In general, the SP phenotype is considered to enrich for chemoresistant tumor cells [2–4]. In agreement, our gene expression analysis of melanoma SP revealed upregulated expression of chemoresistance genes (see Table 1 and Table S5 in File S1). Therefore, we tested the effect of dacarbazine (DTIC) - the current standard chemotherapeutic agent for melanomas [23] -, in the A375 cell line. First, time- and dose-response of cell toxicity was determined (Figure S3 in File S2). Cell death was most prominent after 3-day exposure (with an IC_50_ of 75µg/ml), in accordance with other reports [34]. A375 cells were then treated during 3 days with double the IC_50_ dose of DTIC (150µg/ml) to obtain a profound toxic effect. As shown in Figure 5A, the SP increased ~10-fold in proportion suggesting that the SP cells are more resistant to DTIC than the non-SP cells.

Gene expression analysis further revealed upregulated expression of anti-apoptotic factors in the SP suggesting resistance to severe conditions such as hypoxia, typically occurring in cancer growth [4,35]. The A375 cell line was grown in 1.5% O_2_ (meaning hypoxia in cell cultures that are standardly kept in 20% O_2_). Analysis of the SP showed a ~45-fold increase in proportion (Figure 5B), indicating that SP cells are more resistant to hypoxic conditions. This finding concurs with the upregulated expression of *EPAS1/HIF2A* in the SP (Figure 4).

### ABCB1^+^ cells are localized in the tumor’s invasive component

**Figure 6:**
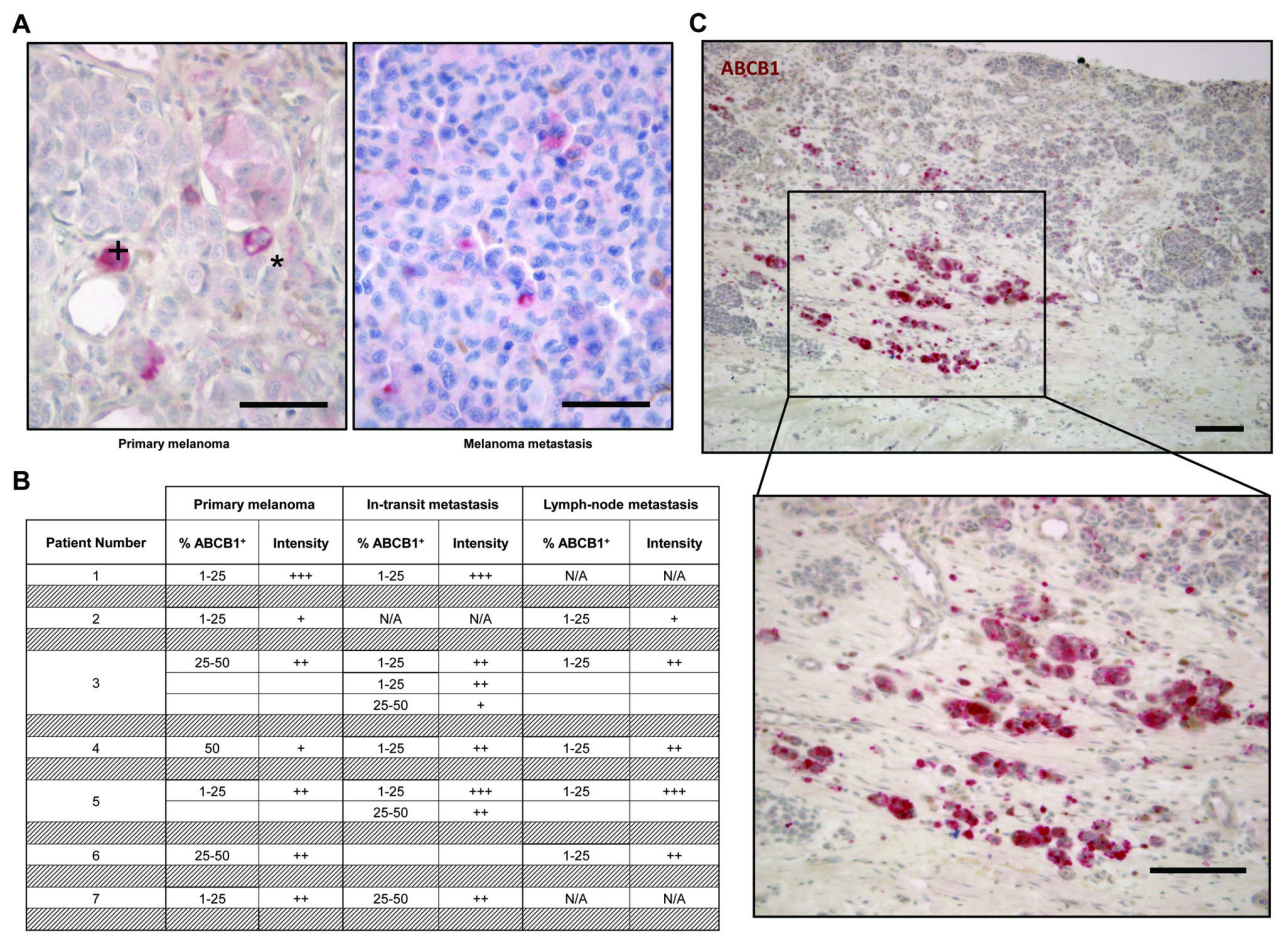
ABCB1 expression in primary human melanomas and corresponding metastases. A) Representative examples of primary melanoma (left) and melanoma metastasis (right) immunostained for ABCB1 (scale bar, 150µm; +, cytoplasmic staining; *, membranous staining). B) Summary of semi-quantitative analysis, showing estimated percentage of ABCB1^+^ cells and signal intensity (no staining = 0, weak signal = +, moderate signal = ++, strong signal = +++; N/A, not applicable). C) Primary melanoma immunostained for ABCB1 (top) and higher magnification of the boxed area (bottom) (scale bar, 300µm).

Gene expression profiling identified upregulated expression of *ABCB1* in the melanoma SP, in agreement with findings by Luo et al. [10]. In order to localize ABCB1-expressing cells in melanomas *in situ*, a series of 20 additional patient tumor samples (7 primary melanomas and 13 corresponding metastases), different from the 38 samples analyzed above, was examined using immunohistochemistry. ABCB1 expression was detected in a subset of melanoma cells, displaying a punctuated cytoplasmic or a membranous staining pattern (Figure 6A). No obvious differences in proportion of ABCB1-immunopositive (ABCB1^+^) were observed between primary melanomas and metastases (Figure 6A-B). Remarkably, ABCB1^+^ cells in the primary melanomas were predominantly found at the invasive front of the tumors (Figure 6C), reminiscent of putative CSC in other cancer types [36]. These ABCB1^+^ cells frequently occurred in small clusters, whereas the ABCB1^+^ cells found scattered within the tumors mostly presented as individual cells. In melanoma metastases, single or clustered ABCB1^+^ cells were only found disseminated over the tumor (and not localized in a front; Figure 6A). In some of the tumors analyzed, similar cells also expressed JARID1B (Figure S4 in File S2). Although low-signal ABCB1 immunoreactivity was also observed in a few endothelial cells and some (tumor-infiltrating) lymphocytes, the tumoral stroma mostly lacked ABCB1 expression. ABCB1 was also absent from most (normal) epidermal cells, although the basal layer of the epidermis showed sporadic immunoreactivity (data not shown).

## Discussion

In the present study, we identified a SP in human melanoma and found molecular and functional indications that this subpopulation is enriched in tumorigenic and chemoresistant cell phenotypes. The chemoresistant character is supported by superior survival under dacarbazine exposure and by upregulated expression of a number of anti-apoptotic (e.g. *BAG4, DDX17* and *MDM2*) and resistance-conferring genes (e.g. *ABCB1*, *SLC18A1* and *MDM2*). The functional basis of resistance to dacarbazine, whether it is for instance due to increased drug efflux, DNA repair or anti-apoptosis, remains to be investigated. We further found that the SP proportion increases under hypoxic conditions supporting the idea that the SP also enriches for melanoma cells that survive harsh conditions occurring in the *in situ* tumor environment. In accordance, *EPAS1/HIF2A*, a hypoxia-inducible factor that enables cancer cells to adapt to low oxygen conditions [37–39], was found upregulated in the melanoma SP.

Our analysis further points to a prognostic potential of the SP proportion as far as primary cutaneous melanomas are concerned, given its correlation with Breslow’s depth [1,24]. Although promising, more samples of primary melanomas will have to be analyzed for thorough support.

The melanoma SP displays tumorigenic capacity that is higher than that of the MP cells in terms of xenograft tumor size, efficiency of tumor formation, number of cells needed for tumor growth and clonogenic activity. Moreover, the SP appears able to re-establish the A375 cell line heterogeneity in culture and can re-form tumors that are histologically comparable to the source melanoma. Because such tumorigenesis as well as chemoresistance and adaptation to aggravating conditions like hypoxia [37] are proposed hallmarks of CSC, the melanoma SP may thus be enriched in CSC or CSC-like cells. Also in other cancer types, the SP has been shown to enrich for CSC(-like) activity (reviewed in 3,4,26–28). In further support, the melanoma SP shows upregulated expression of factors that by others have been assigned to (candidate) melanoma CSC, including *ABCB1, DNMT3B, EPAS1, JARID1B* and *TERT* [8,13,22]. These factors may also have important functions in melanoma biology and therapy resistance. The DNA methyltransferase DNMT3B maintains hematopoietic and neural stem cells, has been shown to promote *in vivo* tumorigenesis of colon cancer by transcriptional silencing of tumor suppressor genes, and is also involved in therapy resistance by inducing cell cycle arrest [40–44]. EPAS1/HIF2A has been proposed as a CSC marker not only in melanoma but also in other cancers like glioma [37,45]. In renal cancer EPAS1 plays an important role in resistance to chemotherapeutics [46]. In addition, EPAS1 is correlated with tumor invasiveness in hepatocellular carcinoma [47]. Interestingly, high expression of EPAS1 has been observed in melanoma with poor prognosis [48]. The telomerase reverse transcriptase TERT promotes stem-like features and drug resistance in both glioma and breast cancer [49,50], and correlates with tumorigenic capacity of melanoma cells [51]. Although the data above were mainly considered within the CSC theory of cancer and support a CSC(-like) phenotype of the melanoma SP, it should be mentioned that the identification of the SP may also fit within the classical clonal evolution model of cancer, with the SP enriching for cells that clonally drive progression of the cancer. In fact, the CSC and clonal evolution theories are not mutually exclusive [52].

In melanoma cell lines, ABCB1 expression has been linked with migration and invasion [53,54]. ABCB1^+^ cells from primary cell cultures of resected melanomas have higher clonogenic capacity than the immunonegative cells [8]. ABCB1 expression has previously been correlated with aggressive melanoma phenotypes, based on Breslow’s depth, Clark’s level and lymph-node involvement [55]. Interestingly, we detected ABCB1 expression in clusters of tumor cells located at the invasive front of the primary melanomas. In colorectal carcinoma, CSC are located in similar tumor regions of expanding and invading borders from where they may eventually migrate and metastasize [36]. Together with the expansion of the melanoma SP in parallel with deeper invasion of the primary tumor (Breslow’s depth), it is tempting to hypothesize that the SP/ABCB1^+^ cells represent CSC(-like cells) that expand and invade during primary melanoma progression, and eventually leave the tumor to migrate to distant sites (metastasis). Of note, ABCB1 immunoreactivity was not only observed in the cell membrane of some melanoma cells but sometimes in the cytoplasm. Intracellular ABCB1 has been shown to play a role in the translocation of drugs like doxorubicin into cytoplasmic vesicles, thereby promoting the drug’s sequestration and efflux [56–58].

The histone lysine demethylase JARID1B has previously been identified in a small subpopulation of melanoma cells, which were characterized as slow-cycling and self-renewing CSC(-like cells) [22]. JARID1B^+^ melanoma cells appeared dispensable for tumor initiation as JARID1B-negative cells were also tumorigenic, but the JARID1B^+^ cells were essential to sustain tumor growth. Along the same line, we here observed that the melanoma SP does not seem to be pivotal for tumor initiation (as also the MP can start xenograft tumors), but that tumors from the SP grow larger. Possibly, the MP – like the JARID1B-negative cell population – may contain rapidly proliferating “cancer progenitor” (transit-amplifying) cells that can initiate a tumor, but that exhaust after a number of cell divisions leading to a lower expansion of the tumor [21,59]. Alternatively, the tumorigenic capacity of the MP may be due to residual contamination of the sorted fraction with a few SP cells. Also in studies relying on membrane markers to sort putative CSC, no absolute purification could be obtained, and the non-CSC compartment also induced tumor growth albeit at (much) lower efficiency than the candidate CSC [21,22,60,61].

ABCB5 has also been advanced as melanoma CSC marker by others [10,18]. However, we (as well as other groups [62]) did not observe consistent but highly variable expression of *ABCB5* in the melanoma SP. Differences in experimental setup may be responsible; Luo et al. [10] studied gene expression of ABC transporters in SP cells of melanoma xenografts whereas our analyses were done on the SP of melanoma samples directly from the patient. In addition, the apparent discrepancy may be due to differences in mRNA and protein expression and stability of ABCB5. Finally, incomplete overlap of proposed melanoma CSC populations has also been observed by others (reviewed in 13).

Further interesting cancer-related genes found upregulated in the melanoma SP include *CXCR4*, *FOSL2, LEF1* and *SOX10*. CXCR4 has previously been detected on a subpopulation of dacarbazine-resistant melanoma cells that play an important role in migration and subsequent metastasis [63]. SOX10 has been implicated in migration and metastasis of B16F10 mouse melanoma cells [64]. LEF1 expression is predominantly observed in melanoma cell lines with high migration capacity, possibly acting through epithelial-mesenchymal transition [65]. And FOSL2 overexpression is associated with a more aggressive and invasive cancer phenotype (as found in breast cancer [66]).

As a final remark, the SP can sometimes co-purify endothelial and immune cells as has been found in some tissues [67]. Our observations so far suggest that this potential contamination does not have a significant influence in our study. First, microarray analysis did not show upregulated endothelial- or immune-related pathways in the melanoma SP *versus* MP. Second, a significant overlap was observed between melanoma SP genes and gene sets upregulated in other cancer cell lines which obviously lack endothelial and immune cells. Third, important genes that were upregulated in the SP of patient melanomas were also found upregulated in the SP of the A375 cell line.

In conclusion, our study provides molecular and functional arguments that the SP from human melanoma is enriched in chemoresistant and tumorigenic cells, and hence may encompass potential CSC. Given these important characteristics, further exploration of the melanoma SP may lead to new insights into melanoma biology and therapy resistance, and eventually to new prognostic markers and therapeutic targets.

## Materials and Methods

### Ethics Statement

In agreement with the admission rules of the University Hospitals, Leuven, Belgium, patients undergoing surgical removal of a primary or metastatic melanoma provided written consent to use their left-over diagnostic material for scientific purposes in an anonymized way; the research project was approved by the local Ethical Committee of the University Hospitals Leuven, and the use of the biological materials was formally approved by the Biobank of the University Hospitals, Leuven. The animal studies were approved by the local Ethical Committee of the KU Leuven.

### Human melanoma specimens

Melanoma samples were obtained after surgical resection in the University Hospitals, Leuven. Clinical information on the samples analyzed (n=38) is shown in Table S1 in File S1. Part of the tumor was used for (immuno-)histological and pathological evaluation, and part (cut into 3-mm^3^ pieces) was dissociated into single cells using collagenase type IV (180U/ml; Life Technologies, Ghent, Belgium) for 2.5 h at 37°C. Cells were subjected to further analysis as described below.

### The human melanoma A375 cell line

The malignant (metastatic) human melanoma cell line A375 [25] was obtained from Dr. L. Van Kempen (Department of Pathology and Oncology, McGill University, Montreal, Canada). Cells were cultured in Dulbecco’s modified Eagle’s medium/Ham’s F12 (DMEM/F12 1:1; Lonza, Verviers, Belgium) supplemented with 4mM L-Glutamine (Life Technologies), 10% fetal bovine serum (FBS; Lonza) and antibiotic/antimycotic (Life Technologies). Cell cultures were regularly tested for mycoplasma contamination by PCR, and were found negative (data not shown). For the analyses described below, cells were released from the culture vessel and dissociated into single cells using trypsin (0.05%) with EDTA (Life Technologies).

### SP analysis by FACS

We adapted the original SP protocol [5] for optimal resolution of SP from human melanoma. Briefly, cell density was adjusted to 1x10^6^ cells/ml and cells were incubated with 5µg/ml Hoechst33342 (Sigma, Bornem, Belgium) for 90 min at 37°C. Verapamil (100µM; Sigma), added 20 min before in control samples, was used to confirm the SP phenotype because it reduces efflux of the dye [5,67]. Cells were finally resuspended in ice-cold PBS (Life technologies) with FBS (2%; Lonza) and propidium iodide (2µg/ml; Sigma) for subsequent analysis by flow cytometry using a FACSVantage or FACSAriaIII (BD Biosciences, Erembodegem, Belgium). The SP is visualized as a Hoechst^low^ population after UV excitation of Hoechst and detection of blue emission with a BP 424/44 filter and of red emission with a BP 630/22 filter. Analysis was done, and proportions calculated, within the living (propidium iodide-negative) cell population. Statistical analysis of SP percentages was performed using one-way ANOVA (Dun’s Multiple Comparison test).

### Exposure to dacarbazine and hypoxia

To obtain time- and dose-response curves of cell toxicity by dacarbazine (DTIC, activated by light; see 68), A375 cells were treated with different concentrations of DTIC (25-200 µg/ml; Sigma) or vehicle (PBS). Viable cells were counted after 1 to 3 days using the 4-methylumbelliferyl heptanoate (MUH) assay (according to [69]). For SP analysis, A375 cells (triplicate) were treated with 150 µg/ml DTIC for 3 days and cells analyzed by FACS as described above.

To investigate the effect of hypoxia on the SP, A375 cells (triplicate) were cultured in 1.5% O_2_/5% CO_2_ using the “In Vivo_2_ 400” hypoxic incubator (Ruskinn Technology, Bridgend, UK) for 3 days, and the SP compared with the SP of A375 cells cultured under standard cell-culture conditions (20% O_2_/5% CO_2_). Statistical analysis of SP percentages was performed using two-tailed unpaired Student’s t-test.

### Culture and colony formation

SP and MP were sorted from subconfluent A375 cell cultures, and equal numbers seeded in culture at 200-1000 cells/ml in the standard culture medium. Both cell populations displayed comparable viability after sorting (Trypan Blue exclusion test; data not shown). Five, 10 and 11 days later, cell cultures were analyzed by FACS to determine the SP proportion.

To assess colony-forming capacity, A375 SP and MP cells were cultured at low density (1000 cells/60-mm dish) in the standard medium. At day 9, cultures were washed twice with PBS, stained for 10 min at room temperature with 1,9-dimethyl-methylene blue (0.25% in 50% ethanol; Sigma) and the number of colonies was counted.

### In vivo tumorigenesis

To assess tumorigenic activity *in vivo*, SP and MP cells from melanoma or A375 were sorted by FACS into DMEM/F12 (supplemented with 10% FBS). Cells were mixed with Matrigel (1:1; BD Biosciences), and varying numbers sc injected into “severe combined immunodeficiency” (SCID) mice (males, 6-10 weeks old; bred in-house). Tumor volume was calculated using the formula [(large side)x(small side) ²]x0.52, and tumor sizes from SP and MP compared at identical time points in the individual experiments.

### Microarray analysis

SP and MP cells from primary (cutaneous malignant) melanomas were sorted by FACS into cold lysis solution of the RNeasy Micro Kit (Qiagen, Venlo, The Netherlands). RNA was extracted according to the manufacturer’s protocol (comprising DNase treatment), and quality and concentration determined using Picochips on a BioAnalyzer 2100 (Agilent Technologies, Diegem, Belgium). Only samples with RNA Integrity Number ≥8.0 were used for microarray analysis (n=4 primary melanomas), which was performed in the VIB Nucleomics Core (Leuven, Belgium). RNA (20ng) was subjected to 2 successive rounds of linear amplification by *in vitro* transcription while incorporating Cy3 label [70]. cRNA probes were hybridized onto Agilent whole human genome 4x44K oligonucleotide arrays (Ag-Hu-WG-G4112F). The microarray data have been deposited in the NCBI’s Gene Expression Omnibus (GEO) and are accessible through GEO Series accession number GSE48838. Data were further processed using the Agilent’s Feature Extraction Software (version 10.1.1.1). Raw signal intensities were background-corrected, subjected to quantile normalization and log_2_-transformed. The obtained data sets of SP and MP were compared in a pair-wise fashion, and p-values calculated by paired Students t-test performed on the log_2_ values of the probe sets. Genes were considered differentially expressed when p<0.05 and fold change≥1.5. To explore the underlying functional categories, clustering analysis of differentially expressed genes was performed using GSEA [71]. Finally, we narrowed the gene sets by focusing on factors shown to be important in “stemness”/CSC, cellular migration, chemoresistance, apoptosis and cell adherence (≥1.5-fold in ≥ half of the samples, p<0.05). To further visualize gene networks, the Search Tool for the Retrieval of Interacting Genes (STRING; http://string-db.org/) was used.

### Reverse-transcription quantitative PCR (RT-qPCR)

For real-time qPCR using SYBR Green, RNA was amplified and converted into cDNA with the Ovation Pico SL WTA kit (NuGEN, Bemmel, The Netherlands). Oligonucleotide primers for qPCR were designed with Perlprimer [72] (see Table S6 in File S1) and validated for comparable amplification efficiency (data not shown). Multiple housekeeping genes were tested for normalization (*GAPDH, HPRT, B2M, RPL19*). Based on the level and stability of expression in all samples (relative to the other housekeeping genes), the most appropriate set of housekeeping genes (i.e. with uniform expression), being *RPL19* and *B2M*, was selected. qPCR was performed on an ABI 7900HT Fast PCR system using Fast SYBR Green Master Mix (Applied Biosystems/Life Technologies) according to the manufacturer’s protocol. Negative controls and dissociation curves were used to confirm specific amplification.

For RT-qPCR using Taqman technology, SP and MP cells were sorted in lysis solution of the Taqman Preamp Cell-to-Ct Kit (Applied Biosystems/Life Technologies) and RNA isolated according to the manufacturer’s protocol (including DNase treatment). After examination of quality and concentration, RNA was amplified and converted to cDNA with the Taqman Preamp Cell-to-Ct Kit. To measure expression of selected genes, corresponding Taqman GEX Assays (containing primers and TaqMan probe) were used (see Table S6 in File S1). *ACTB* was included as housekeeping gene for normalization, as recommended by the manufacturer. qPCR was performed on an ABI 7900HT Fast PCR system using Taqman Gene Expression Master Mix (Applied Biosystems/Life Technologies) following the manufacturer’s recommendations.

Normalized, relative expression data were calculated using the comparative threshold cycle (2^ddCt^) method [73]. Statistical analysis was performed using two-tailed one sample t-test (if Gaussian distributed) or two-tailed Wilcoxon rank sum test.

### Immunohistochemistry

Five-µm sections of formalin-fixed paraffin embedded melanoma specimens were immunostained using a Bond-max fully automated staining system (Leica Microsystems, Wetzlar, Germany), including onboard heat-induced antigen retrieval during 20 min in EDTA-Tris buffer, pH 9.0 (for ABCB1) or citrate buffer, pH 6.0 (for JARID1B) and alkaline phosphatase-based Bond Polymer Refine Red Detection. The primary antibodies were used at optimized dilutions, i.e. 1/200 for ABCB1 (Clone JSB-1; MON9011 from Monosan/Sanbio, Uden, The Netherlands) and 1/100 for JARID1B (HPA027179 from Sigma). Specificity of the ABCB1 antibody [74] has been amply validated before in our lab, using multiple types of normal tissues and tumors [75–77]. The anti-JARID1B antibody [78] was manufactured by the Human Protein Atlas (HPA), which validates the affinity-purified antibodies by immunohistochemistry in a multitude of tissues and cells assembled in tissue microarrays, by immunofluorescence and confocal microscopy in human cell lines, by Western blot and by protein array analyses (http://www.proteinatlas.org/ [79]). In addition, we routinely perform immunohistochemistry using these antibodies on control specimens (human tissue with known expression pattern such as liver for ABCB1 and skin for JARID1B) with positive results.

Sections were evaluated for estimated abundance of immunopositive cells and signal intensity (no staining = 0, weak signal = +, moderate signal = ++, strong signal = +++) by 2 investigators. 

## Supporting Information

File S1 **Table S1.** Clinical information on the 38 melanoma samples analyzed. ^1^F, female; M, male^2^. SSMM, superficial spreading malignant melanoma^3^. NA, not available. **Table S2.** Differentially expressed genes in SP *versus* MP as identified by microarray analysis of primary melanomas. ^1^Genes differentially expressed between SP and MP in 4 primary CMM as analyzed by microarray (≥1.5-fold, p<0.05). Genes differentially expressed in ≥3 of the 4 tumors samples are indicated in bold. ^2^Fold up- or downregulation in SP versus MP (average of the 4 patient samples, Pts 1-4). **Table S3.** GSEA of genes upregulated in the SP *versus* MP of primary melanomas. ^1^Gene Set Enrichment Analysis (GSEA) of the 142 human ENSEMBL IDs upregulated in the SP versus MP in ≥3 of the 4 microarrayed primary CMM (≥1.5-fold, p<0.05). **Table S4.** GSEA of genes downregulated in the SP *versus* MP of primary melanomas. ^1^Gene Set Enrichment Analysis (GSEA) of the 109 human ENSEMBL IDs downregulated in the SP versus MP in ≥3 of the 4 microarrayed primary CMM (≥1.5-fold, p<0.05). **Table S5.** Additional information on the genes mentioned in Table **1**. ^1^References to the literature of the differentially expressed genes selected for Table 1 as related to "stemness"/CSC, therapy resistance, apoptosis, metastasis, and cell adherence, invasion and migration. ^2^PMID, PubMed Identifier. **Table S6.** Summary of functionally interesting genes analyzed by RT-qPCR in SP *versus* MP from primary melanomas and A375. [1]Function of the selected genes as related to ABC transporters (chemoresistance) and "stemness"/CSC. [2]Corresponding references to literature. [3]Sequences of the oligonucleotide primers used for qPCR. [4]Taqman GEX Assays (containing primers and TaqMan probe) used for qPCR. [5]PMID, PubMed Identifier (NA, Not Applicable) **Table S7.** Microarray expression data of genes found significantly upregulated in the melanoma SP *versus* MP by RT-qPCR. ^[1]^Fold up- or downregulation in SP *versus* MP (average of the 4 primary melanoma samples as analyzed by microarray).
(XLSX)

File S2 **Figure S1.** Microarray expression data of human melanoma SP versus MP: concise validation by RT-qPCR and interaction network by STRING analysis. A) Expression ratios (SP/MP) determined by RT-qPCR of a few interesting genes that were found upregulated in the SP in microarray analysis; RT-qPCR was performed on the limited residual RNA/cDNA of 2 of the microarrayed melanoma samples. B) STRING analysis of genes upregulated in the human melanoma SP versus the MP, displayed as “evidence view” (i.e. only connected nodes are shown). **Figure S2.** Overview of functionally interesting genes not significantly upregulated in the melanoma SP. Expression ratios of the indicated genes related to ABC transporters and CSC markers, in the SP versus the MP from 3 primary melanomas and 4 melanoma metastases, as analyzed by RT-qPCR. **Figure S3.** Time- and dose-response curves of dacarbazine toxicity on A375 cells. Cells were treated with different doses of dacarbazine (DTIC) for 1, 2 or 3 days, and cell viability (relative to control) analyzed using the 4-methylumbelliferyl heptanoate (MUH) assay. **Figure S4.** JARID1B expression in primary human melanoma. Primary melanoma immunostained for JARID1B (top) and higher magnification of the boxed area (bottom) (scale bar, 300µm).
(PDF)
