# Supplementary figures and images for: The Human Melanoma Side Population Displays Molecular and Functional Characteristics of Enriched Chemoresistance and Tumorigenesis
Source: PLoS One. 2013 Oct 3;8(10):e76550. doi: 10.1371/journal.pone.0076550 (PMC3789681; doi:10.1371/journal.pone.0076550)

# Figure S1

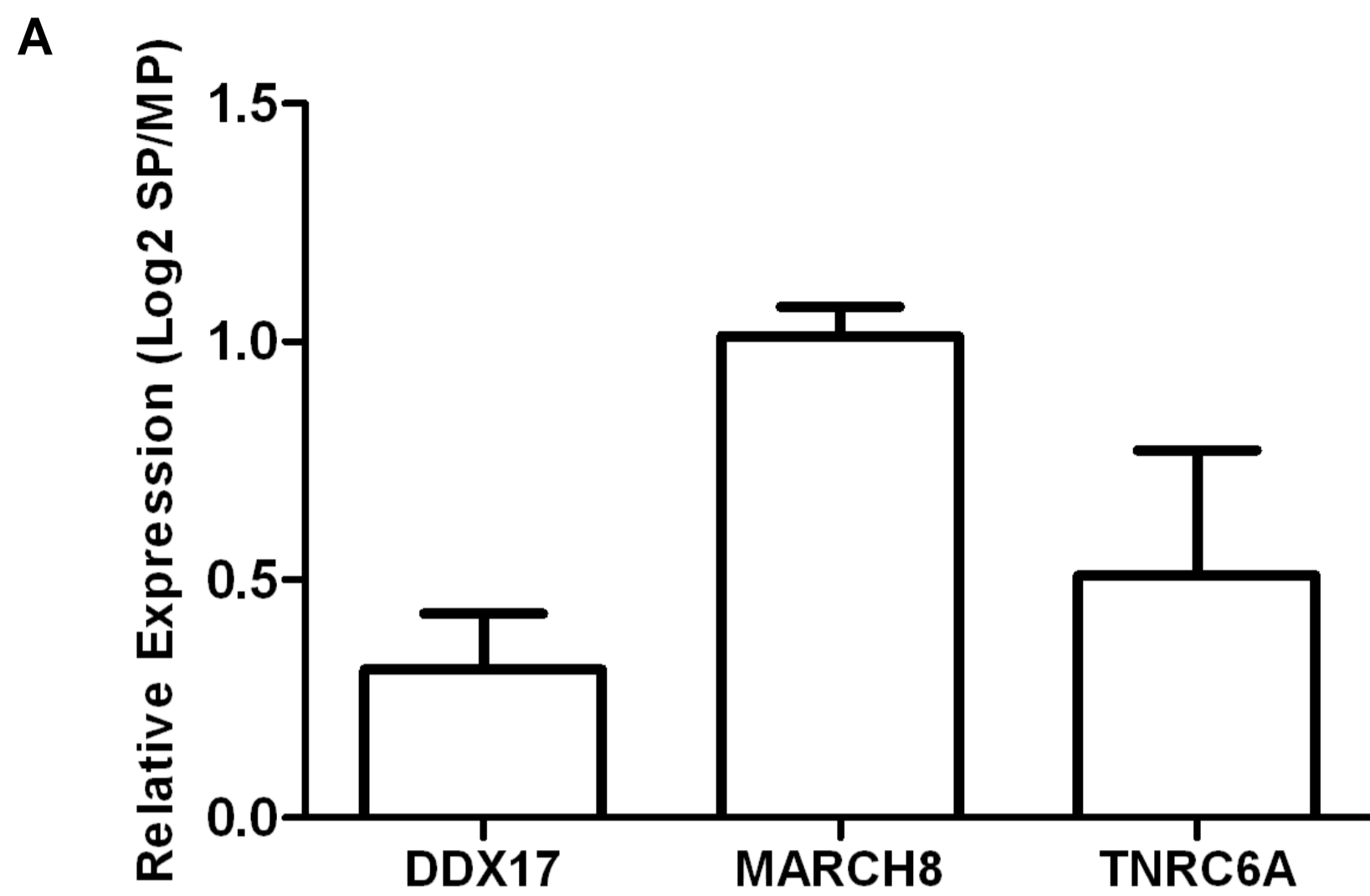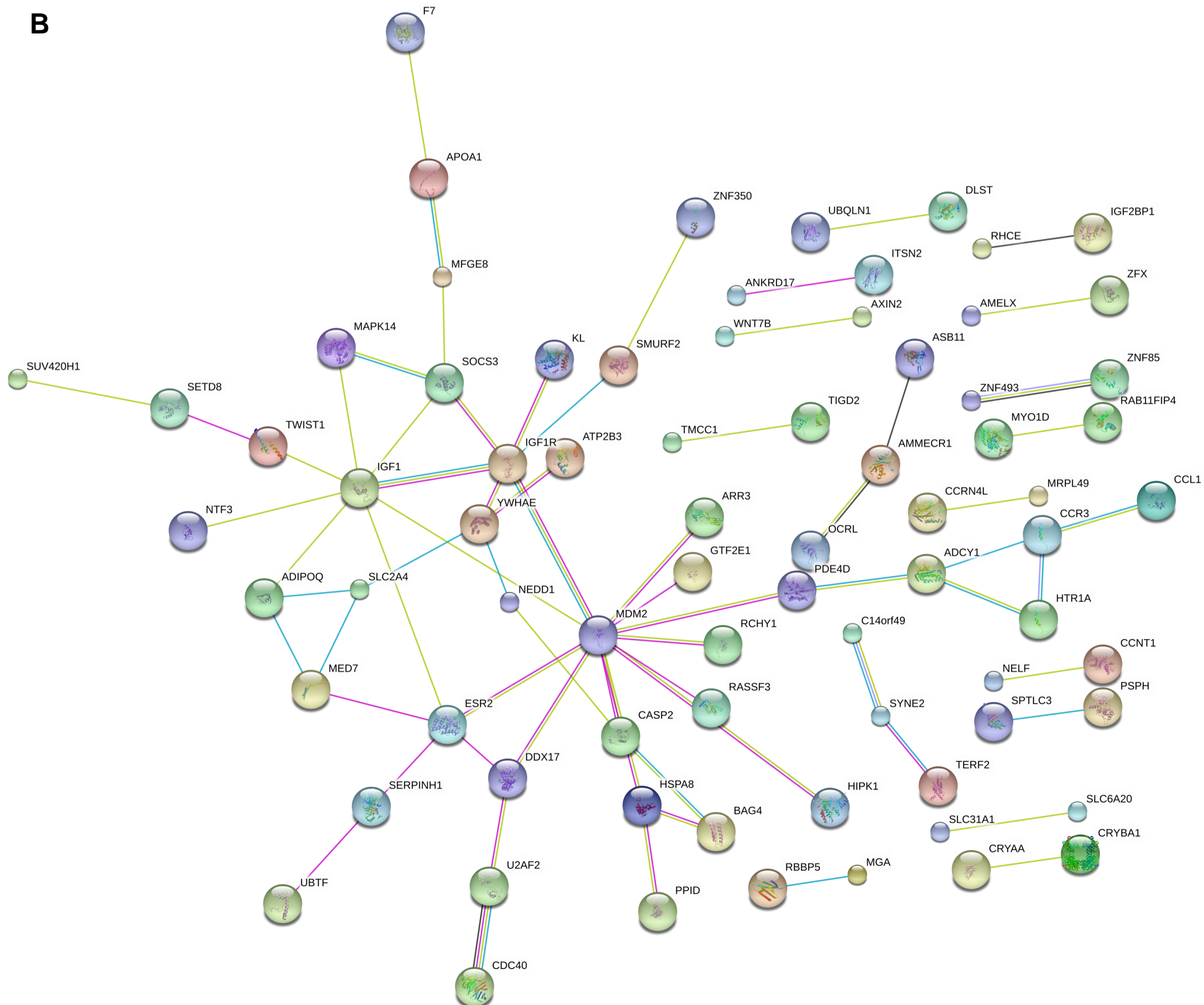

Figure S2

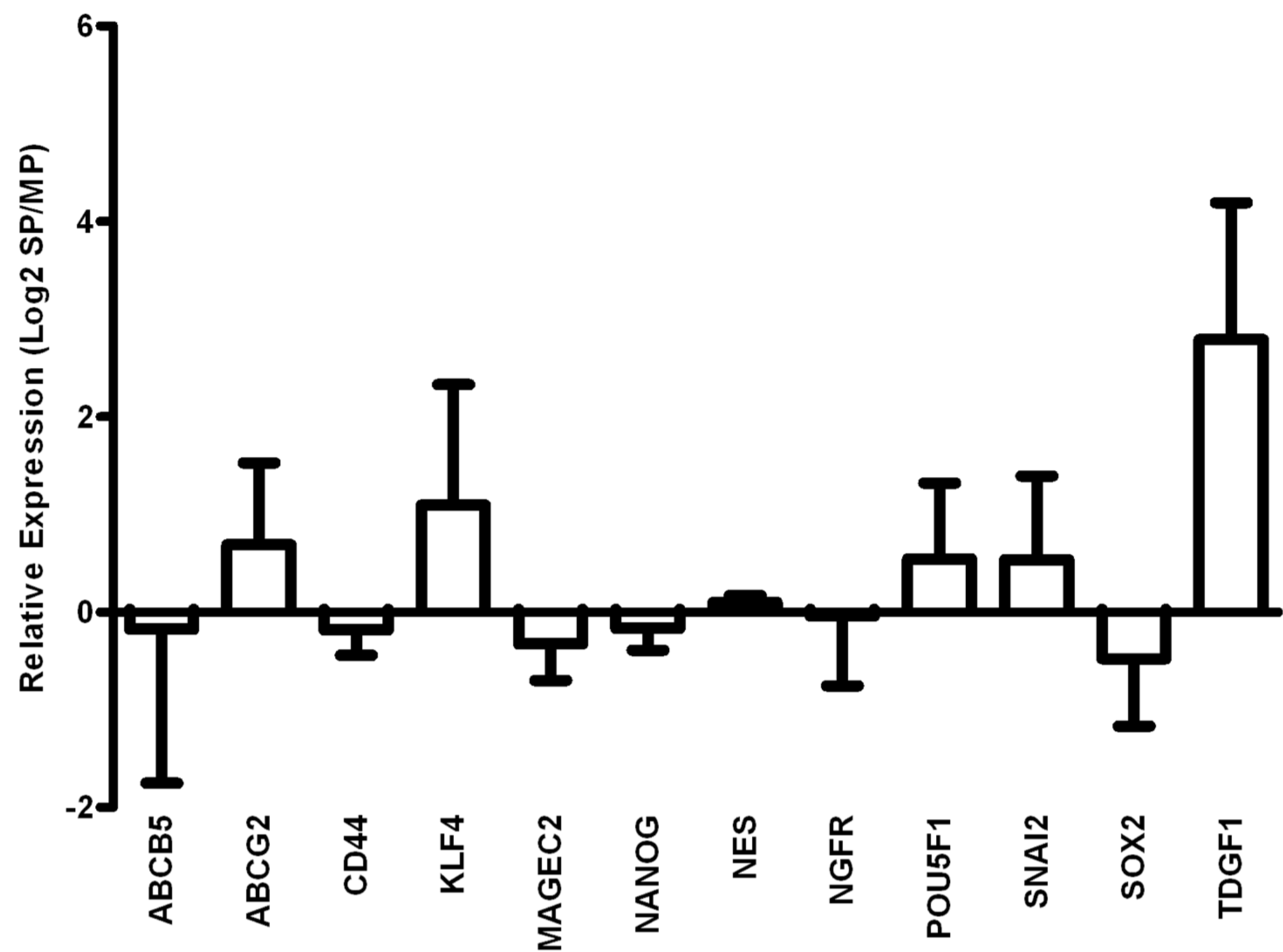

Figure S3

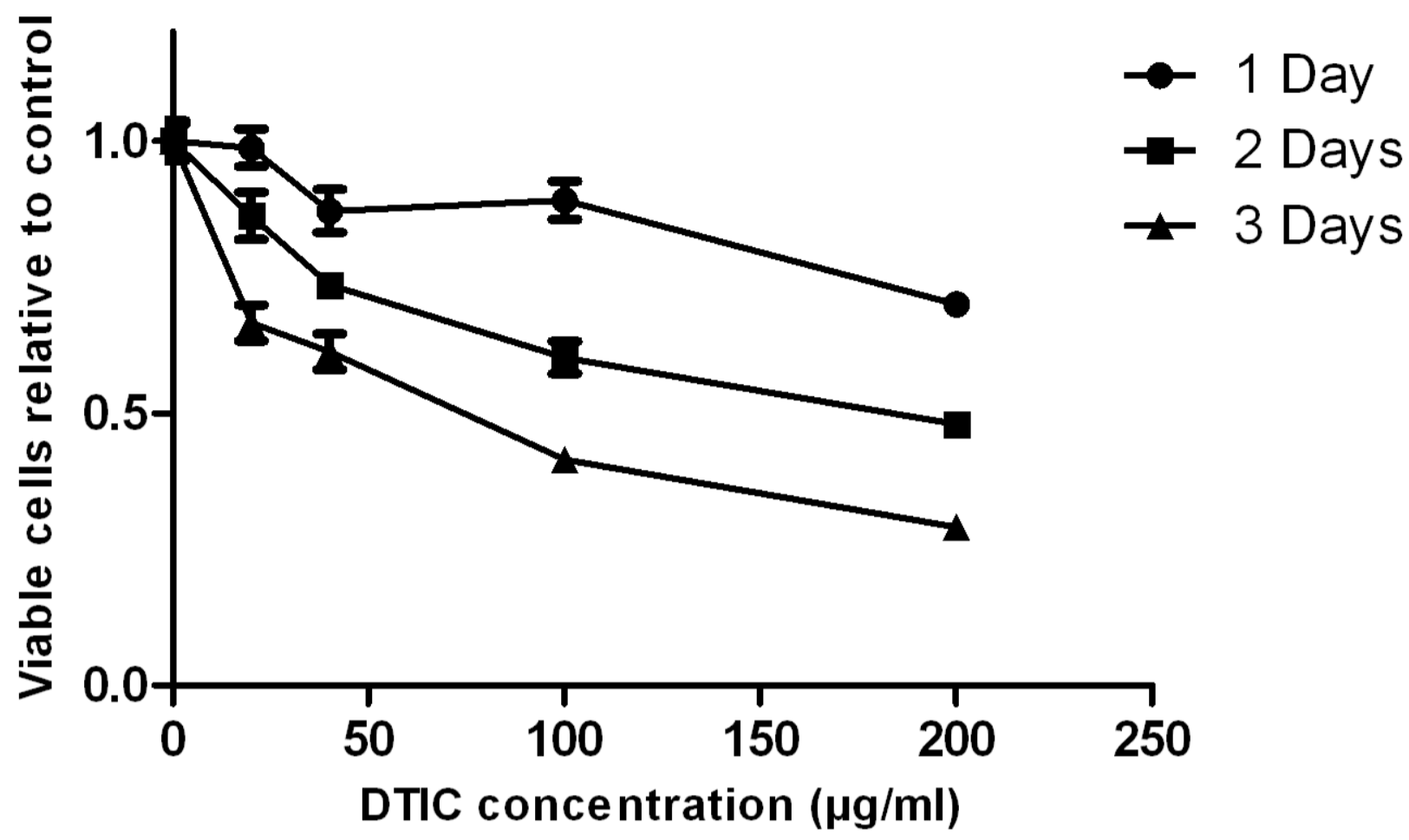

Figure S4

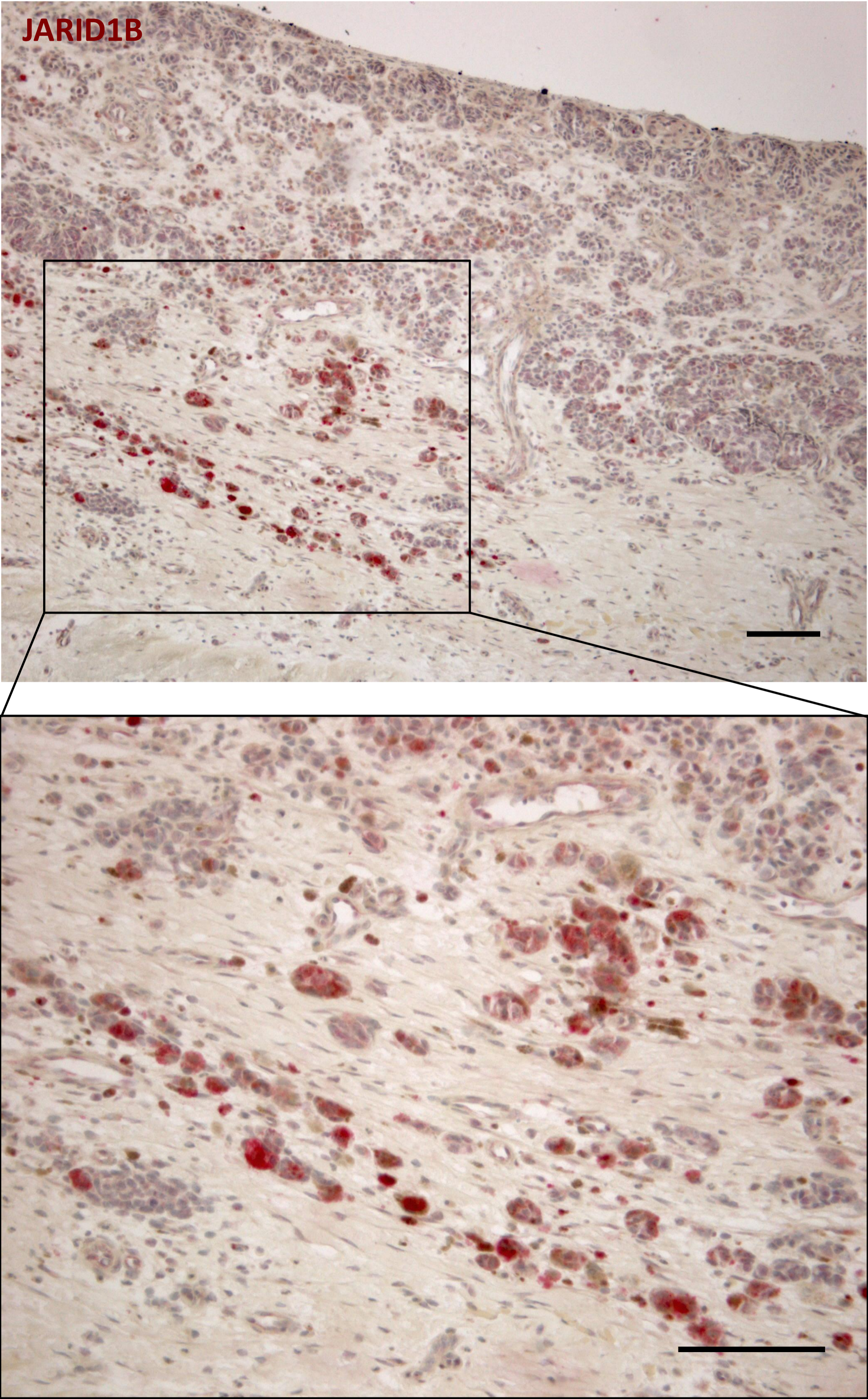

Supplement: File S2 — Figure S1. Microarray expression data of human melanoma SP versus MP: concise validation by RT-qPCR and interaction network by STRING analysis. A) Expression ratios (SP/MP) determined by RT-qPCR of a few interesting genes that were found upregulated in the SP in microarray analysis; RT-qPCR was performed on the limited residual RNA/cDNA of 2 of the microarrayed melanoma samples. B) STRING analysis of genes upregulated in the human melanoma SP versus the MP, displayed as “evidence view” (i.e. only connected nodes are shown). Figure S2. Overview of functionally interesting genes not significantly upregulated in the melanoma SP. Expression ratios of the indicated genes related to ABC transporters and CSC markers, in the SP versus the MP from 3 primary melanomas and 4 melanoma metastases, as analyzed by RT-qPCR. Figure S3. Time- and dose-response curves of dacarbazine toxicity on A375 cells. Cells were treated with different doses of dacarbazine (DTIC) for 1, 2 or 3 days, and cell viability (relative to control) analyzed using the 4-methylumbelliferyl heptanoate (MUH) assay. Figure S4. JARID1B expression in primary human melanoma. Primary melanoma immunostained for JARID1B (top) and higher magnification of the boxed area (bottom) (scale bar, 300µm). (PDF) [file pone.0076550.s009.pdf]
